# Assessment of water quality and occurrence of multidrug-resistant clinically relevant bacteria in drinking water in the twin cities of Pakistan

**DOI:** 10.1007/s10661-025-13989-5

**Published:** 2025-04-12

**Authors:** Nudrat Nadeem, Muhammad Nadeem, Nazish Bostan, Sadia Sattar, Roger Simm, Sundus Javed

**Affiliations:** 1 Microbiology and Public Health Laboratory, Department of Biosciences, COMSATS University Islamabad, Islamabad, Pakistan; 2 Molecular Virology Laboratory, Department of Biosciences, COMSATS University Islamabad, Islamabad, Pakistan; 3 Department of Biosciences, University of Oslo, Oslo, Norway

**Keywords:** Water quality, Water filtration plant, Multidrug resistance, Cephalosporin, Cefotaxime, Heavy metals

## Abstract

Water filtration plants are a main source of drinking water for local populations in almost all big cities of Pakistan but these filtration plants are ineffective in eliminating contaminants from water. This study was conducted to evaluate water quality with specific focus on physicochemical parameters and bacterial contamination including occurrence of multidrug-resistant bacteria in drinking water collected from filtration plants (*N* = 64) of the twin cities of Pakistan. Physicochemical parameters were analyzed following American Public Health Association standard guidelines and total coliform count was determined using membrane filtration method. Gram-negative bacteria were isolated and characterized in terms of species and antibiotic susceptibility. Elevated levels of total dissolved solids, nitrates, total coliforms, lead and copper were found in several samples. Multivariate correlation coefficient revealed significant positive correlation between nitrate levels and total coliform count in bacteriologically contaminated samples. Antibiotic resistance patterns revealed multidrug-resistant and extensively drug-resistant bacteria with high resistance against third generation cephalosporins. Our results highlight the urgent need for improved water filtration processes and regular monitoring to provide safe drinking water to the local community.

**Supplementary Information:**

The online version contains supplementary material available at 10.1007/s10661-025-13989-5.

## Introduction

Waterborne illnesses contribute to half of the global infectious disease burden (Wen et al., 2020). In Pakistan, poor water quality from both ground and surface water is a major health concern and it is estimated that 40% of all diseases and about 30% of all deaths among all age groups occur due to consumption of poor-quality water (Butt & Khair, 2016). Waterborne illnesses contribute to a national GDP loss of 0.6–1.44% that equates to a yearly sum of 380–884 million USD (Rashed & Muhammed, 2021). Antimicrobial resistance is another public health concern and the AMR burden in Low and Middle Income Countries is high due to poor sanitation, inadequate hygienic measures, and overuse and misuse of antimicrobials in human and animal sectors (Ayukekbong et al., 2017). The presence of antimicrobial-resistant (AMR) bacteria in drinking water can lead to gastrointestinal and extraintestinal infections that can be challenging to treat. Waterborne AMR bacteria may also facilitate the transfer of resistance to the gut microbiome, creating a reservoir of AMR and potentially multidrug-resistant (MDR) bacteria (Anthony et al., 2021). Illnesses caused by AMR bacteria are difficult to manage, often resulting in prolonged hospital stays and higher treatment costs.

Provincial, federal and local government have installed drinking water filtration plants in most major cities of Pakistan, providing the primary source of drinking water. The Islamabad, Rawalpindi metropolitan area is the country’s third-largest metropolitan area and a significant portion of the population of 3.4 million relies on drinking water filtration plants to fulfill their drinking water needs (Sohail et al., 2020). Efficacy of these drinking water filtration plants is dependent on maintenance, operation and cleanliness practices that are often neglected and water quality from these filtration plants is a major concern. Water purification technologies use conventional methods like filtration that cannot remove all the pollutants effectively and completely (Hasan & Muhammad, 2020). Membrane filters used in filter cartridges of drinking water filtration plants, trap contaminants and serve as reservoirs of biological and chemical contaminants that can be released in drinking water if the filters are not replaced timely.

Common contaminants found in drinking water are bacteria, viruses, protozoa, organic and inorganic pollutants, and different heavy metals that require special methods and techniques for detection (Sharma & Bhattacharya, 2017). Environmental factors like pH, temperature and heavy metals can impose selective pressure on microorganisms (Arshad et al., 2017) and alter their cell physiology. High concentration of heavy metals can co-select for AMR bacteria (Chen et al., 2015).

The aim of this study was to assess water quality and evaluate potential correlations between water quality parameters and occurrence of pathogenic and AMR bacteria in drinking water from water filtration plants in the Twin Cities of Pakistan. We first determined different water quality parameters and then investigated possible correlations among physicochemical parameters, heavy metals and coliform counts from drinking water samples. We isolated clinically important bacteria including *Escherichia coli*, *Vibrio cholera* and *Providencia alcalifaciens* from these samples and determined the phenotypic and genotypic patterns of antibiotic resistance.

## Methodology

### Study area and sampling

Samples were collected during the spring and summer months Mar–Jun 2022 from the region of Islamabad and Rawalpindi commonly known as the twin cities of Pakistan. Tap water samples were collected from the filtration plants used for public consumption. For analysis of physical parameters non-sterile 1500-ml Polyethylene plastic bottles were used to collect drinking water. Bottles were first rinsed 2–3 times with tap water from the filtration plant and then sample was collected. For biological water quality assessment (total coliform count) and isolation of bacteria, 100-ml drinking water was collected in clean sterile bottles from the tap of the filtration plant. Separate samples were collected for heavy metal analysis, where 40-ml drinking water was collected in clean sterile 50-ml falcon tubes containing 100 µl of concentrated HNO_3_. Sample collection containers were properly labelled and transported to the Microbiology and Public Health Laboratory at the Biosciences Department of COMSATS University, Islamabad. All drinking water samples were collected from filtration plants installed by the Capital Development Authority (CDA) and Water and Sanitation Agency (WASA) in Islamabad and Rawalpindi, respectively. A total of *N* = 64 drinking water samples were collected from Islamabad (*n* = 32) and Rawalpindi (*n* = 32).

### Physicochemical parameters

Physicochemical parameters including Electrical conductivity (EC), total dissolve solids (TDS), pH and turbidity were evaluated at the time of sample collection. EC and TDS were analyzed using the TDS meter 2.0 (Health Metrics, USA). The pH was determined using an HI99192 drinking water pH meter (Hanna Instrument, Italy), and the turbidity was analyzed with a 2020we/wi portable turbidity meter (LaMotte, USA). Total hardness, alkalinity, Calcium (Ca^2+^), Magnesium (Mg^2+^), Sodium (Na^+^), Potassium (K^+^), Chloride (Cl^−^), Fluoride (F) and Nitrates were measured using American Public Health Association (APHA) guidelines (Association, 1998). Total coliform count was done using membrane filtration method (Method 9132, USEPA; (USEPA, 1978). Heavy metals Cadmium (Cd), Lead (Pb), Chromium (Cr), Nickel (Ni), Copper (Cu), Zinc (Zn) and Iron (Fe) were detected and quantified using atomic absorption spectrometry (iCE 3400, Thermoscientific). 1000-ppm stock solutions (Scharlau Chemical SA, Barcelona, Spain) were used to prepare standard solutions (0.2, 0.4, 0.6, 0.8 and 1 ppm) and calibration curves were plotted and used as reference.

### Water quality assessment

Water quality index (WQI) was calculated using a weighed arithmetic water quality index method as previously described by Brown and co-workers (Brown et al., 1970, 1972).

Heavy metal pollution index (HPI) is a rating technique that imparts the individual composite influence of each heavy metal on overall drinking water quality. HPI was calculated using a previously described method (Aslam et al., 2020).

The formulas used for calculation of WQI (1) and HPI (2) are identical, but the parameters used are different. For simplicity, we refer to the score calculated using Eq. (1) as WQI throughout the manuscript.

1 $$WQI=\frac{{\sum }_{i=1}^{n}WiQi}{{\sum }_{i=1}^{n}Wi}$$

2 $$HPI=\frac{{\sum }_{i=1}^{n}WiQi}{{\sum }_{i=1}^{n}Wi}$$

where:

3 $$Qi=100\times \frac{Vi-V0}{Si-V0}$$

4 $$Wi=\frac{K}{Si}$$

5 $$K=\frac{1}{{\sum }_{i=1}^{n}\frac{1}{Si}}$$

where Wi is the unit weight, Qi represents the sub-index value, Si is the standard value, Vi is the measured value, V0 is the optimal value, which is zero for all heavy metals and 7 for pH (USEPA-NPDWG, 2022; USEPA-NSDWG, 2022). K is a proportionality constant.

In this study, 6 parameters were selected for WQI calculations including pH, TDS, Cl, F, nitrates and total coliform count.

The parameters chosen for the HPI calculations were Pb, Cd, Cr, Ni, Cu, Zn, and Fe. The USEPA recommended standards for drinking water were used (USEPA-NPDWG, 2022; USEPA-NSDWG, 2022).

The critical HPI score is 100, above this value, drinking water is unsuited for consumption.

### Bacterial isolation, identification and antibiotic resistance

*Vibrio cholera* was isolated by adding 1-ml water sample in 9-ml alkaline peptone water and incubated for 6–8 h at 41 °C. 10 ul from the supernatant was then streaked on TCBS (Oxoid-Hampshire, UK) agar and plates were incubated for 24 h at 37 °C. Total coliform count was performed using membrane filtration method using M-endo agar (Liofilchem, Italy) as described earlier (Method 9132, USEPA; (USEPA, 1978). Gram-negative bacteria were isolated using MacConkey agar (Oxoid, UK) and identification was done using Matrix assisted laser desorption/ionization time of flight (MALDI-TOF) mass spectrometry. A single bacterial colony was suspended in a solution containing the matrix α-Cyano- 4-hydroxycinnamic acid (CHCA). Ten ul of the bacterial/matrix mixture was then spotted on a MALDI sample plate. The sample plate was loaded into the MALDI-TOF MS instrument (bioMerieux, France) and results for bacterial fingerprinting and identification were analyzed through the SARAMIS database. Identification of *V. cholera* was performed using a specific primer set for the conserved OMPW gene (Supplementary Table 1). CLSI guidelines were followed for phenotypic culture sensitivity testing using disc diffusion (CLSI, 2021). Antibiotics used were Penicillin (piperacillin:100 µg), Nitrofurans (Nitrofurantoin: 300 µg), Cephalosporin (Cefepime 30 µg, Cefotaxime: 30 µg, Cefoperzone 75 µg and Ceftazidime 30 µg), Carbapenems (Imipenem: 10 µg), Aminoglycosides (Gentamicin: 10 µg, Amikacin: 30 µg), Quinolones (Ciprofloxacin: 5 µg), Folate antagonist (Cotrimoxazole: 25 µg) and Fosfomycin: 200 µg. All antibiotics were purchased from Oxoid, UK. *E. coli* (BL21) was used as a reference strain for antibiotic susceptibility testing.

### Genotypic antibiotic resistance

Genotypic antibiotic resistance was detected against beta lactamase groups A, B and D in clinically important MDR *Enterobacteriaceae* and non*-Enterobacteriaceae* including 4 *E. coli*, *1 P. alcalifaciens* and 2 V*. cholera* isolates. Genotypic screening of beta lactamase was performed using specific sets of primers for class A, class B and class D beta lactamase groups through conventional PCR. DNA was extracted using one tube genomic DNA extraction kit (Bio basic, USA). Oligonucleotide primers and PCR conditions are described in Supplementary Table 2.

### Statistical analyses

GraphPad and Microsoft Excel 2010 were used to analyze the data using multivariate Pearson’s correlation coefficient matrix and regression analysis, and heatmap was plotted to check the possible correlation among different physicochemical parameters, *p* < 0.05 was considered statistically significant. Linear regression was plotted from nitrate and total coliform count to determine potential correlation of nitrate levels with bacterial contamination.

## Results and discussion

### Physicochemical parameters and drinking water quality index

Physicochemical parameters of drinking water provide essential insights into its condition and suitability for human consumption. Prolonged chemical contamination of drinking water may result in adverse health effects. The United States Environmental Protection agency (USEPA) standards were used for the comparison of physicochemical water quality parameters. (USEPA-NPDWG, 2022; USEPA-NSDWG, 2022). Most of the measured physicochemical parameters were below the permissible limits (Table 1) and turbidity was lower than the detection limit in most of the samples (data not shown).

**Table 1:** Summary of water quality parameters in Islamabad and Rawalpindi

Parameter	Islamabad (*n* = 32)		Rawalpindi (*n* = 32)		Limits
	Mean ± SD	Range	Mean ± SD	Range	
TDS (mg/L)	358 ± 93	217–545	453 ± 153	196–891	500**
pH	7.49 ± 0.22	7.01–8.0	7.5 ± 0.2	7.0–8.0	6.5–8.5**
Alkalinity (mg/L)	270 ± 76	115–361	328 ± 67	130–460	Not regulated
EC (ms)	548 ± 187	239–923	795 ± 240	346–1485	Not regulated
Hardness (mg/L)	281 ± 58	180–370	337 ± 70	170–465	Not regulated
Cl (mg/L)	19.3 ± 7.6	9–46	33.7–14.8	14–79	250**
Na (mg/L)	27.8 ± 13.1	9–57	48.4 ± 30.9	16–145	Not regulated
K (mg/L)	4.49 ± 0.47	3.8–5.5	4.69 ± 0.48	4.2–6.4	Not regulated
Ca (mg/L)	73 ± 17	42–98	89.4 ± 16.5	45–125	Not regulated
Mg (mg/L)	25.0 ± 9.1	9–46	32.3 ± 13.7	8–66	Not regulated
F (mg/L)	0.34 ± 0.12	0.15–0.57	0.77 ± 0.34	0.04–1.41	4*
Nitrate (mg/L)	6.6 ± 2.8	3–13.2	7.0 ± 2.6	3.8–13	10*
Pb (mg/L)	0.0079 ± 0051	0–0.021	0.009 ± 0.0054	0.002–0.021	0.015*
Cd (mg/L)	0.0021 ± 0.00056	0.001–0.0035	0.00211 ± 0.007	0.001–0.0037	0.005*
Cr (mg/L)	0.022 ± 0.011	0.0016–0.054	0.024 ± 0.016	0.006–0.073	0.1*
Ni (mg/L)	0.019 ± 0.015	0.004–0.075	0.035 ± 0.018	0.006–0.079	0.1***
Cu (mg/L)	0.86 ± 0.53	0.22–2.3	0.96 ± 0.59	0–2.3	1.3*
Fe (mg/L)	0.126 ± 0.19	0.008–0.99	0.66 ± 0.55	0.017–2.41	5**
Zn (mg/L)	0.118 ± 0.057	0.03–0.23	0.144 ± 0.058	0.049–0.26	0.3**

Permissible limits of each parameter according to the United States Environmental Protection agency (USEPA 2022) National Primary Drinking Water Regulations (NPDWG, USEPA 2022)*, National Secondary Drinking Water Regulations (NSDWR, USEPA, 2022)**,USEPA 1995 ***

TDS and nitrate levels were found to be higher than the permissible limits in some samples (Table 1). The range of measured concentrations of lead and copper spanned above and below the permissible limits indicating that some but not all samples contained elevated levels of these heavy metals. This correlates with PCRWR water monitoring reports from Islamabad and Rawalpindi from 2010 and 2013, respectively, which revealed that most of the physicochemical parameters from drinking water filtration plants were within the normal reference values (Pakistan Council of Research in Water Resources, 2010, 2013).

**Fig. 1:**
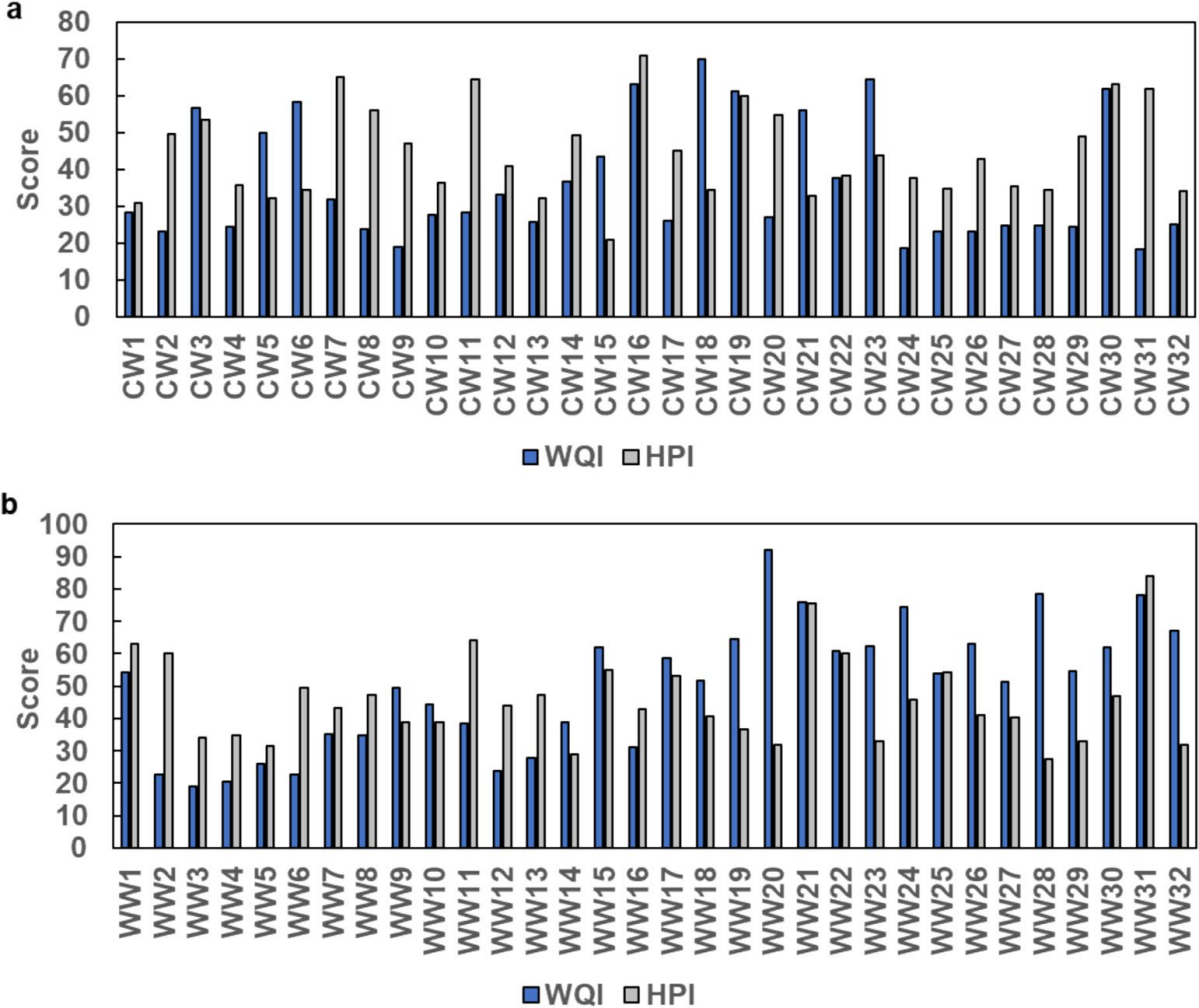
Assessment of water quality parameters from Islamabad (**a**) and Rawalpindi (**b**). Blue and grey bars represent WQI and HPI respectively. For WQI a score above 50 represents poor water quality and a score above 75 very poor water quality. For HPI scores above 100 the water is unsuited for consumption. CW and WW represent the designations of samples from Islamabad and Rawalpindi, respectively. WQI, Water Quality Index; HPI, Heavy metal Pollution Index

WQI was calculated to describe and compare the overall water quality status of the water filtration plants. The selection of WQI parameters was based on region-specific factors that can influence both ground and surface water in twin cities and which are of public health concern. Monitoring of WQI helps in making informed decisions whether the water is safe for human consumption or needs purification. 25% of samples from Islamabad and 44% of samples from Rawalpindi showed a WQI-score (51–75) indicating poor quality (Fig. 1). 12% of samples from Rawalpindi were classified as very poor (Fig. 1b).

These data show that drinking water from the Rawalpindi area were of poorer quality in general compared to the drinking water from Islamabad. Rawalpindi lies within densely populated zones, suggesting that population expansion and urbanization are major factors in deterioration of drinking water quality (ul Haq et al., 2021). Our data is generally consistent with a previous study conducted from the same study area, published in 2020 by Sohail et al. (Sohail et al., 2020). Fewer samples in our study had very poor or unsuitable drinking water quality compared to the study by Sohail et al. (Sohail et al., 2020). This is likely due to the choice of parameters and potentially due to seasonal variations and different time points for sampling. However, the data indicating poorer water quality in Rawalpindi compared to Islamabad is consistent over time.

HPI calculated based on lead, cadmium, chromium, nickel, copper, zinc and iron showed that the HPI score was below the HPI critical value (HPI = 100) for all water samples (Fig. 1). The heavy metals cadmium, chromium, nickel, zinc, and iron were also found to be within safe limits. However, 12.5% of the samples were contaminated with lead and 23.4% of samples showed slightly higher concentration of copper compared to the recommended USEPA standards. Our results for lead contamination were somewhat lower than a previous study conducted in the twin cities where 17.21% of sampled sites were found to be contaminated with lead (Rana et al., 2022). It is not known if this is a real improvement over time or natural variation due to differences in environmental conditions at the sampling times. Possible sources of the lead contamination are leaching of water pipes and air pollution from burning of lead containing fuels (Barn et al., 2013; Tracy et al., 2020). Lead is a toxic heavy metal that can cause a range of symptoms in humans including gastrointestinal and neurological disorders and children suffering from lead poisoning often present with cognitive disabilities (Wani et al., 2015). High copper levels further highlight an emerging concern that needs immediate attention by regulatory authorities. Prolonged exposure to elevated copper levels can cause gastrointestinal symptoms and liver damage (Rafati Rahimzadeh et al., 2024). Among the samples from 64 monitored plants from Islamabad and Rawalpindi, 45% were found to be unsafe as a consequence of bacterial contamination and 14% were found to be unsafe due to excessive nitrate levels. For 32.8% of the samples, all measured physicochemical and biological measurements met the criteria for safe drinking water.

### Relationship among water quality parameters

**Fig. 2:**
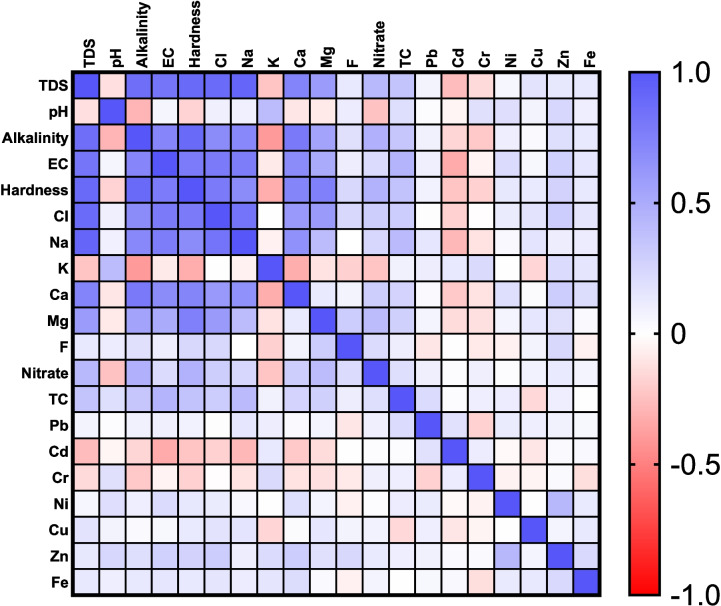
Heatmap from multivariate Pearson’s correlation coefficient analysis showing relationship between physicochemical parameters from drinking water samples. Dark blue color indicates strong positive correlation and dark pink color indicates strong negative correlation. TDS, Total Dissolved Solids; EC, Electrical Conductivity; TC, Total Coliform count

Pearson’s multivariate correlation coefficient analysis was performed to determine possible correlations among different parameters in water samples and a heatmap was plotted (Fig. 2).

A strong positive correlation was observed between water hardness and TDS and further revealed a positive correlation with different cations and anions including Ca^2+^, Na^+^, Mg^2+^, and Cl^−^. Previous publications have also reported high levels of calcium and magnesium in the study area and a correlation to water hardness (Shabbir & Ahmad, 2015; Sohail et al., 2020; Tauseef Azam et al., 2024). The concentration of calcium and magnesium in drinking water locations of the twin cities have previously been linked to geological mineral deposits (Tauseef Azam et al., 2024). High levels of TDS in water may result in mineral deposition like calcium carbonate and scaling formation on filtration equipment and particularly on reverse osmosis (RO) membranes resulting in reduced efficacy and short life span of the filtration equipment (Soti & Gupta, 2021). Moreover, seasonal and site-specific factors like underlying aquifer composition and local pollution sources can collectively influence water quality parameters (Zhang et al., 2019).

**Fig. 3:**
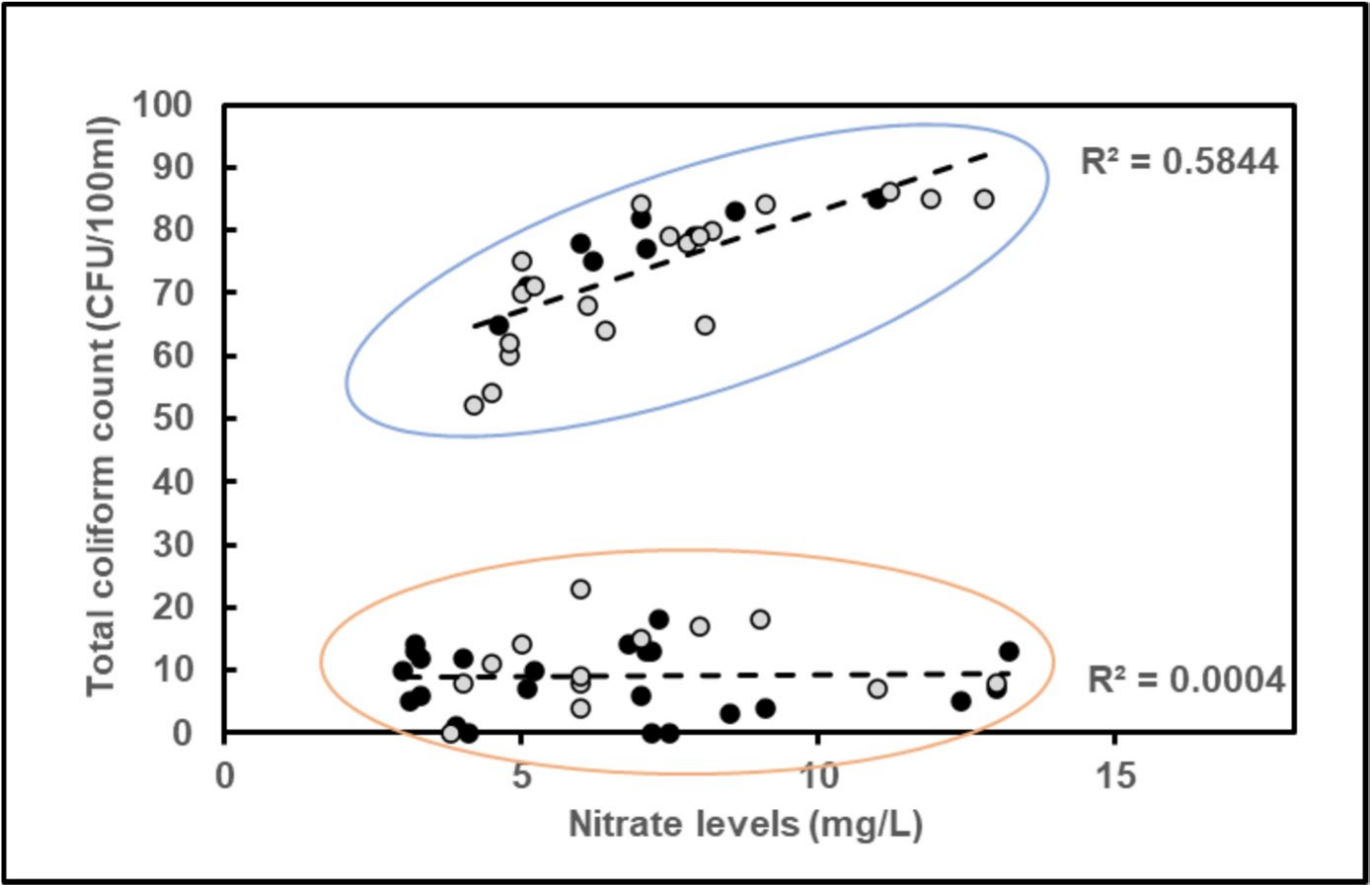
Correlation of total coliform count and nitrate levels from all sampling sites. Black dots show samples from Islamabad and grey dots refer to samples from Rawalpindi. Blue and red ovals highlight two distinct populations of samples with high and low total coliform counts, respectively. The population of highly contaminated samples show a linear correlation between total coliform count and nitrate concentration. There is no correlation between total Coliform count and nitrate levels in samples with low total coliform counts

There was a weak positive correlation between total coliform counts and most physicochemical parameters, except for potassium, fluoride, cadmium and iron. Plotting total coliform count against nitrate levels revealed two populations among the sampling sites. One population contained samples with high total coliform counts and one population constituted samples with low total coliform counts. Linear regression analysis of the two populations showed that there is a correlation between total coliform counts and nitrate concentration in samples from bacteriologically contaminated study sites (Fig. 3). There was no correlation between bacterial load and nitrate levels in samples containing low total coliform counts.

The water quality from the twin cities varied between sampling sites and were significantly contaminated by nitrates and microorganisms. Similar findings from the twin cities (Daud et al., 2017) shows that this contamination is persistent. In 2021, PCRWR reported bacteriological and nitrate contamination in drinking water from almost all big cities of Pakistan that highlights the common occurrence of these contaminants and need of attention (Rashed & Muhammed, 2021). Nitrates can have direct negative health effects, especially on pregnant women and small children (Clemmensen et al., 2023; Ward et al., 2018). Positive correlation between nitrate levels and microbial contamination in water suggests a common source or underlying interactive mechanism for these contaminants. Both nitrate and microbial contaminants can originate from agricultural runoff, and human sewage systems which are likely sources for contamination in the drinking water of the twin cities. This dual contamination requires a multi-faceted approach for mitigation, including regular monitoring of nitrate and microbial levels, identifying and reducing contamination sources, and implementing advanced purification regimens to effectively remove both contaminants.

### Occurrence of clinically relevant bacteria and analysis of antibiotic resistance

**Fig. 4:**
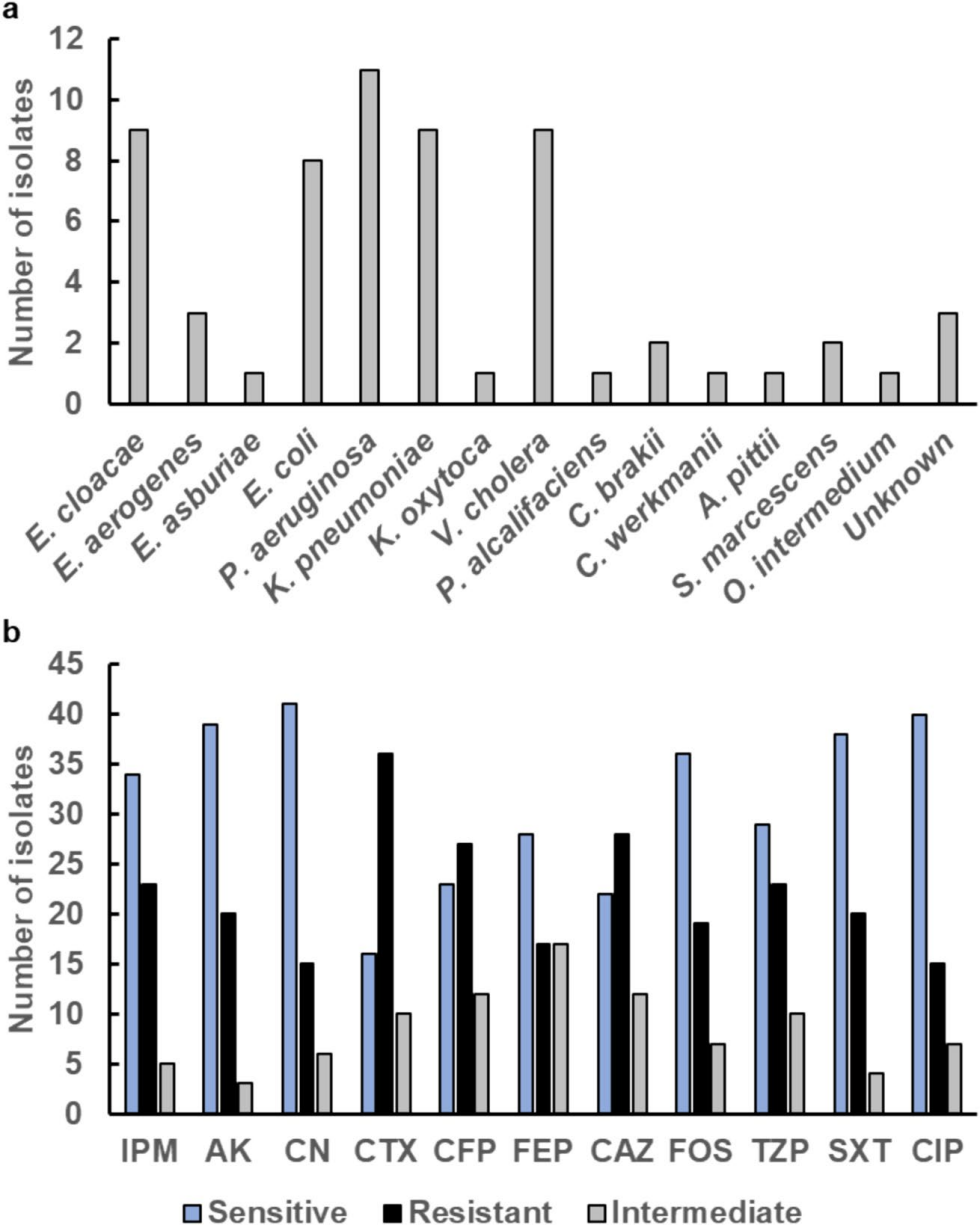
Characterization of bacteria isolated from drinking water. **A** Occurrence of bacterial species among isolates from drinking water (**B**) Antibiotic susceptibility among bacterial isolates from drinking water. Antibiotic susceptibility was determined using disc diffusion and clinical break points for *Enterobacteriaceae* were used for assigning isolates as sensitive, intermediate or resistant. IPM: Imipenem, AK: Amikacin, CN: Gentamicin, CTX: Cefotaxime, CFP: Cefoperazone, FEP: Cefepime, CAZ: Ceftazidime, FOS: Fosfomycin, TZP: Tazobactam, SXT: Co-trimoxazole, CIP: Ciprofloxacin

Previous reports have generally focused on the total coliform and fecal coliform contamination from drinking water filtration plants (Pakistan Council of Research in Water Resources, 2010, 2013). However, there is a deficiency of systematic investigations of diversity and distribution of bacterial species and occurrence of AMR in drinking water from filtration plants. In this study, bacterial isolation and identification revealed high prevalence of opportunistic pathogens in drinking water samples obtained from bacteriologically contaminated areas. Sixty-two Gram negative bacterial isolates were cultured from 29 bacteriologically contaminated water samples and species were identified using MALDI TOF. *V. cholera*, was further identified based on the presence of the *ompW* gene through PCR as previously described (Jiang et al., 2018). More than half of the bacterial isolates (53%) were identified as *Klebsiella pneumoniae, Pseudomonas aeruginosa* and *Enterobacter* spp. (Fig. 4a) belonging to the ESKAPE pathogens (*Enterococcus faecium, Staphylococcus aureus, Klebsiella pneumonia, Acinetobacter baumannii, Pseudomonas aeruginosa, and Enterobacter *spp.*)* which are a group of bacteria responsible for the majority of nosocomial infections worldwide. These bacteria pose a health risk to the vulnerable community especially children, immunocompromised individuals and the elderly (LeChevallier et al., 2024). 50% of all bacterial isolates were multidrug resistant (MDR) and another 17.7% were extensively drug resistant (XDR). None of the isolates were pan resistant, but one isolate of *P. aeruginosa* was resistant to 9 of 11 tested antibiotics. Overall resistance against beta lactam antibiotics was observed and 58% of the isolates were resistant to the cephalosporin Cefotaxime (Fig. 4b).

Seven clinically important MDR isolates were selected and screened for class A, class B and class D beta lactamase genes. Five of these isolates were positive for *bla*_*CTX-M*_, that confers resistance against Cefotaxime and 1 isolate each was positive for *bla*_*SHV*_ and *bla*_*TEM*_. From the class B beta lactamase group, only 1 isolate was positive for *bla*_NDM_, *bla*_VIM_, *bla*_SIM_ and *bla*_GIM_ genes whereas 2 isolates were positive for *bla*_SPM_ and *bla*_IPM_. One isolate carried the *bla*_OXA-51_ and *bla*_OXA-24_ genes and 2 isolates were positive for *bla*_OXA-23_. The class D beta lactam gene *bla*_OXA-58_ was detected in 3 isolates. The most prevalent beta lactamase gene in this study was *bla*_CTX-M_. Our findings from the genotypic antibiotic resistance patterns revealed *Enterobacteriaceae* isolates harboring group A, B and D beta lactamase genes and none of the beta lactam genes was found in *V. cholera*. Identification of beta lactamase genes in drinking water isolates were also reported by Xi et al. (Xi et al., 2009) who investigated the occurrence, prevalence and dynamics of antibiotic resistance genes in bacteria from treated drinking water. In 2018, a meta-analysis was performed from Pakistan to determine the prevalence of ESBL producing *Enterobacteriaceae* and most commonly found genes were *bla*_*TEM*_, *bla*_*CTX*_, *bla*_*SHV*_ and *bla*_*OXA*_ (Abrar et al., 2018) that highlights the significant occurrence of beta lactamase genes in Pakistan. Another study reported that most prevalent beta lactamase genes in 15 ESBL *E. coli* isolates from water samples were *bla*_*TEM*_*bla*_*CTX-M*_ (Aitezaz Ahsan et al., 2022a, 2022b). A longitudinal surveillance study conducted in 2019 across various Pakistani hospitals, including those in the twin cities, revealed that the most frequently prescribed antibiotics were cephalosporin. Third-generation cephalosporins accounted for 78.1% of cephalosporin prescriptions. High usage rate of third generation cephalosporins is a potential driver for the development of AMR and dissemination of ESBL producing bacteria in environmental and clinical settings (Saleem et al., 2019). Differential distribution of beta lactamase genes in *Enterobacteriaceae* but not in *V. cholera* is consistent with their ecological dynamics and genetic adaptability where acquiring beta lactamase resistance may confer a survival disadvantage to *V. cholera* in aquatic environments. Moreover, *V. cholerae* comprises the *var* regulon that encodes a metallo beta lactamase and antibiotic efflux system regulated by a LysR-type transcription factor and confers resistance to penicillins, cephalosporins and carbapenems (Lin et al., 2017), which reduces the requirement for acquiring additional beta lactam resistance mechanisms..

Contamination of drinking water sources in highly populated areas with MDR enteric pathogens is a big threat to human health (Delgado-Gardea et al., 2016; Kumar et al., 2013) and also contribute towards the global antimicrobial resistance crisis. In Pakistan treatment of the diarrheal infections depends upon resources availability and severity of diarrheal infection. Broad spectrum antibiotics especially third generation cephalosporins and fluroquinolones are often prescribed empirically due to limited diagnostic facilities (Centre for Disease Dynamics, Economics & Policy, 2018). Use of these broad-spectrum antibiotics may not only give rise to antibiotic resistant bacteria but also promote their release from clinical setups into the environment and other resources. AMR bacteria have previously been detected in surface water, wastewater and sewage from the Twin cities (A. Ahsan et al., 2022a, 2022b; Yasmin et al., 2022; Zahra et al., 2018), and antibiotics have been detected in wastewater (Zafar et al., 2021) suggesting dissemination of these contaminants between hospitals, the environment and the community. There is an urgent need to address the AMR challenge in Pakistan with a One health approach. Regular monitoring of AMR bacteria in drinking water and improved maintenance routines of water filtration plants are important steps in reducing development and spread of AMR.

## Conclusion

Our study along with the previous studies demonstrate persistently poor drinking water quality from the filtration plants of Islamabad and Rawalpindi over time. Membrane filters in filtration plants are designed to remove most contaminants; however, our results show that they are currently ineffective at removing lead, copper, nitrate and bacteria from drinking water. Poor water quality from several filtration plants renders the drinking water unfit for human consumption. There is an urgent need for improved routines regarding monitoring of the drinking water quality and maintenance of the water filtration plants. To assess the emerging threat of antibiotic-resistant bacteria in drinking water, continuous monitoring should be integrated into the water safety protocols.

## Supplementary Information

Below is the link to the electronic supplementary material.

Supplementary file 1 (DOCX 25.5 KB)

## Data Availability

Data is provided within the manuscript or supplementary information files.
