# Supplementary material for: Assessment of water quality and occurrence of multidrug-resistant clinically relevant bacteria in drinking water in the twin cities of Pakistan
Source: Environ Monit Assess. 2025 Apr 12;197(5):543. doi: 10.1007/s10661-025-13989-5 (PMC11993497; doi:10.1007/s10661-025-13989-5)
Supplement: Supplementary file 1 — (DOCX 25.5 KB) [file 10661_2025_13989_MOESM1_ESM.docx]

**Assessment of Water Quality and Occurrence of Multidrug Resistant Clinically Relevant bacteria in Drinking Water in the Twin Cities of Pakistan**

**Nudrat Nadeem^1,3^, Muhammad Nadeem^1^, Nazish Bostan^2^, Sadia Sattar^2^, Roger Simm^3*^, Sundus Javed^1^**

^1^ COMSATS University Islamabad, Microbiology and public health Laboratory, Department of Biosciences, Islamabad, Pakistan

^2^ COMSATS University Islamabad, Molecular Virology Laboratory, Department of Biosciences, Islamabad, Pakistan

^3^ University of Oslo, Department of Biosciences, Oslo, Norway

*** Corresponding author**

Email: [roger.simm@ibv.uio.no](mailto:roger.simm@ibv.uio.no)

ORCID ID:

Roger Simm 0000-0001-5187-2456

**Supplementary Information**

| **Supplementary Table 1.** Water quality classification Based on WQI score* | | |
| --- | --- | --- |
| **Category** | **WQI value** | **Quality status** |
| I | <25 | Excellent |
| II | 26-50 | Good |
| III | 51-75 | Poor |
| IV | 76-100 | Very poor |
| V | >100 | Unsuitable |
| WQI, Water Quality Index (Brown, McClelland et al. 1970, Brown, McClelland et al. 1972). | | |

| **Supplementary Table 2**. Primer sets for Bacterial identification and detection of beta lactam antibiotic resistance genes in bacterial isolates | | | | |
| --- | --- | --- | --- | --- |
| **Gene** | **Primer sequence** | **PCR conditions** | **Product size** | **Reference** |
| OMPW | F: CACCAAGAAGGTGACTTTATTGTG  R: GAACTATAACCACCCGCG | Initial denaturation (95 ͦC/5min)  Denaturation (95 ͦ C/1min)  Annealing (55 ͦ C/90s)  Extension (72 ͦ C/90s)  Final extension (72 ͦ C/10min)  Cycles: 30 | 586bp | (Jiang, Bi et al. 2018) |
| 16SRNA | 27F: AGAGTTTGATCCTGGCTC AG  1392R: GGTTACCTTGTTACGACTT | Initial denaturation (95 ͦC/3min)  Denaturation (95 ͦ C/30s)  Annealing (50 ͦ C/45s)  Extension (72 ͦ C/90s)  Final extension (72 ͦ C/7min)  Cycles:35 | 1492bp | (Chen, Guo et al. 2019) |
| **Class A Beta lactamase genes** | | | |  |
| CTXM | F: TTTGCGATGTGCAGTACCAGTAA  R: CGATATCGTTGGTGGTGCCATA | Initial denaturation (95 ͦ C/5min)  Denaturation (95 ͦ C/30s)  Annealing (51 ͦ C/50s)  Extension (72 ͦ C/60s)  Final extension (72 ͦ C/10min)  Cycles:30 | 544bp | (Ahmed, Omar et al. 2013) |
| SHV | F: TGTTAGCCACCCTGCCGCT  R: GTTGCCAGTGCTCGATCAG | Initial denaturation (95 ͦ C/3min)  Denaturation (95 ͦ C/30s)  Annealing (60 ͦ C/45s)  Extension (72 ͦ C/60s)  Final extension (72 ͦ C/5min)  Cycles: 30 | 825bp | (Huang, Liu et al. 2014) |
| TEM | F: GAGTATTCAACATTTCCGTGTG  R: TAATCAGTGAGGCACCTATCTC | Initial denaturation (95 ͦ C/2min)  Denaturation (95 ͦ C/60s)  Annealing (58 ͦ C/30s)  Extension (72 ͦ C/60s)  Final extension (72 ͦ C/5min)  Cycles:35 | 848bp | (Sirous, Hashemzadeh et al. 2020) |
| **Class B Beta lactamase genes** | | | |  |
| NDM | F: GGTTTGGCGATCTGGTTTTC  R: CGGAATGGCTCATCACGATC | Initial denaturation (95 ͦ C/5min)  Denaturation (95 ͦ C/40s)  Annealing (52 ͦ C/50s)  Extension (72 ͦ C/30s)  Final extension (72 ͦ C/5min)  Cycles:30 | 621bp | (Poirel, Walsh et al. 2011) |
| VIM | F: GATGGTGTTTGGTCGCATA  R: CGAATGCGCAGCATCCAG | Initial denaturation (95 ͦ C/5min)  Denaturation (95 ͦ C/40s)  Annealing (52 ͦ C/50s)  Extension (72 ͦ C/30s)  Final extension (72 ͦ C/5min)  Cycles:30 | 390bp | (Poirel, Walsh et al. 2011) |
| SIM | F: TACAAGGGATTCGGCATCG  R: TAATGGCCTGTTCCCATGTG | Initial denaturation (95 ͦ C/5min)  Denaturation (95 ͦ C/40s)  Annealing (52 ͦ C/50s)  Extension (72 ͦ C/30s)  Final extension (72 ͦ C/5min)  Cycles:30 | 570bp | (Poirel, Walsh et al. 2011) |
| IMP | F: GGAATAGAGTGGCTTAAYTCT  R: CCAAACYACTASGTTATCT | Initial denaturation (95 ͦ C/5min)  Denaturation (95 ͦ C/40s)  Annealing (52 ͦ C/50s)  Extension (72 ͦ C/30s)  Final extension (72 ͦ C/5min)  Cycles:30 | 188bp | (Ellington, Kistler et al. 2007) |
| SPM | F: AAAATCTGGGTACGCAAACG  R: ACATTATCCGCTGGAACAGG | Initial denaturation (95 ͦ C/5min)  Denaturation (95 ͦ C/40s)  Annealing (52 ͦ C/50s)  Extension (72 ͦ C/30s)  Final extension (72 ͦ C/5min)  Cycles:30 | 271bp | (Poirel, Walsh et al. 2011) |
| GIM | F: TCGACACACCTTGGTCTGAA  R: AACTTCCAACTTTGCCATGC | Initial denaturation (95 ͦ C/5min)  Denaturation (95 ͦ C/40s)  Annealing (52 ͦ C/50s)  Extension (72 ͦ C/30s)  Final extension (72 ͦ C/5min)  Cycles:30 | 477bp | (Poirel, Walsh et al. 2011) |
| **Class D beta lactam primers** | | | |  |
| OXA23 | F: GATCGGATTGGAGAACCAGA  R: ATTTCTGACCGCATTTCCAT | Initial denaturation (95 ͦ C/5min)  Denaturation (95 ͦ C/30s)  Annealing (52 ͦ C/40s)  Extension (72 ͦ C/50s)  Final extension (72 ͦ C/6min)  Cycles:30 | 501bp | (Chen, Guo et al. 2019) |
| OXA24 | F: GGTTAGTTGGCCCCCTTAAA  R: AGTTGAGCGAAAAGGGGATT | Initial denaturation (95 ͦ C/5min)  Denaturation (95 ͦ C/30s)  Annealing (52 ͦ C/40s)  Extension (72 ͦ C/50s)  Final extension (72 ͦ C/6min)  Cycles:30 | 246bp | (Chen, Guo et al. 2019) |
| OXA 51 | F: TAATGCTTTGATCGGCCTTG  R: TGGATTGCACTTCATCTTGG | Initial denaturation (95 ͦ C/2min)  Denaturation (95 ͦ C/60s)  Annealing (54 ͦ C/30s)  Extension (72 ͦ C/60s)  Final extension (72 ͦ C/5min)  Cycles:30 | 353bp | (Sirous, Hashemzadeh et al. 2020) |
| OXA 58 | F: AAGTATTGGGGCTTGTGCTG  R: CCCCTCTGCGCTCTACATAC | Initial denaturation (95 ͦ C/5min)  Denaturation (95 ͦ C/30s)  Annealing (52 ͦ C/40s)  Extension (72 ͦ C/50s)  Final extension (72 ͦ C/6min)  Cycles:30 | 599bp | (Chen, Guo et al. 2019) |

**References:**

Ahmed, O. B., A. O. Omar, A. H. Asghar, M. M. Elhassan, A.-M. Al-Munawwarah and S. Arabia (2013). "Prevalence of TEM, SHV and CTX-M genes in Escherichia coli and Klebsiella spp Urinary Isolates from Sudan with confirmed ESBL phenotype." Life Sci J **10**(2): 191-195.

Brown, R. M., N. I. McClelland, R. A. Deininger and M. F. O’Connor (1972). A water quality index—crashing the psychological barrier. Indicators of Environmental Quality: Proceedings of a symposium held during the AAAS meeting in Philadelphia, Pennsylvania, December 26–31, 1971, Springer.

Brown, R. M., N. I. McClelland, R. A. Deininger and R. G. Tozer (1970). "A water quality index-do we dare." Water and sewage works **117**(10).

Chen, Y., P. Guo, H. Huang, Y. Huang, Z. Wu and K. Liao (2019). "Detection of co-harboring OXA-58 and NDM-1 carbapenemase producing genes resided on a same plasmid from an Acinetobacter pittii clinical isolate in China." Iranian Journal of Basic Medical Sciences **22**(1): 106.

Ellington, M. J., J. Kistler, D. M. Livermore and N. Woodford (2007). "Multiplex PCR for rapid detection of genes encoding acquired metallo-β-lactamases." Journal of antimicrobial chemotherapy **59**(2): 321-322.

Huang, S.-R., M.-F. Liu, C.-F. Lin and Z.-Y. Shi (2014). "Molecular surveillance and clinical outcomes of carbapenem-resistant Escherichia coli and Klebsiella pneumoniae infections." Journal of Microbiology, Immunology and Infection **47**(3): 187-196.

Jiang, F., R. Bi, L. Deng, H. Kang, B. Gu and P. Ma (2018). "Virulence-associated genes and molecular characteristics of non-O1/non-O139 Vibrio cholerae isolated from hepatitis B cirrhosis patients in China." International Journal of Infectious Diseases **74**: 117-122.

Poirel, L., T. R. Walsh, V. Cuvillier and P. Nordmann (2011). "Multiplex PCR for detection of acquired carbapenemase genes." Diagnostic microbiology and infectious disease **70**(1): 119-123.

Sirous, M., M. Hashemzadeh, M. Keshtvarz, M. Amin, N. Shams, M. Dastoorpoor, M. Shahin and D. Koraei (2020). "Molecular characterization and antimicrobial resistance of enteropathogenic Escherichia coli in Children from Ahvaz, Iran." Jundishapur Journal of Microbiology **13**(7).
